# 

**Published:** 1899-05-27

**Authors:** 


					THE HOSPITAL. "The standard of highest purity."? Lancet, -11AI I,I'll, lawi.
CADBURY'S rf8* H OADBURY'S O
n cocoa )% MM JP cocoa
^ - ABSOLUTELY PURE. ^ NO DRUGS OR ALKALIES USED.
ORWICH HoSPITia
? ULfKSCOrj /red I C rt /? i/*rin/nnni
No. 661. Vol. XXVI. [aFe^aper!] SATURDAY,
May 27ih, 1899.
[Subscriptionjenns,] pRICE TWOPENCE.

				

